# Epidemiology of Carbapenem-Resistant Enterobacteriaceae at a Long-term Acute Care Hospital

**DOI:** 10.1093/ofid/ofy224

**Published:** 2018-10-03

**Authors:** Teena Chopra, Christopher Rivard, Reda A Awali, Amar Krishna, Robert A Bonomo, Federico Perez, Keith S Kaye

**Affiliations:** 1 Division of Infectious Diseases, Detroit Medical Center, Wayne State University School of Medicine, Detroit, Michigan; 2 Division of Infectious Diseases and HIV Medicine, Louis Stokes Cleveland VA Medical Center, Case Western Reserve University School of Medicine, Cleveland, Ohio

**Keywords:** horizontal transmission, *Klebsiella pneumoniae* Carbapenemase (KPC), multilocus sequence typing, repetitive sequence–based PCR

## Abstract

**Background:**

Residents of long-term acute care hospitals (LTACHs) are considered important reservoirs of multidrug-resistant organisms, including Carbapenem-resistant Enterobacteriaceae (CRE). We conducted this study to define the characteristics of CRE-infected/colonized patients admitted to an LTACH and the molecular characteristics of the CRE isolates.

**Methods:**

This retrospective study was conducted to collect information on demographic and comorbid conditions in CRE-colonized/infected patients admitted to a 77-bed LTACH in Detroit between January 2011 and July 2012. Data pertaining to hospital-related exposures were collected for 30 days before positive CRE culture. Polymerase chain reaction (PCR) gene amplification, repetitive sequence–based PCR, and multilocus sequence typing (MLST) were performed on 8 of the CRE isolates.

**Results:**

The study cohort included 30 patients with CRE-positive cultures, 24 (80%) with infections, and 6 (20%) with colonization. The mean age of cohort was 69 ±12.41 years; 19 (63%) patients were ventilator-dependent, and 20 (67%) were treated with at least 1 antibiotic. Twenty-three (77%) patients had CRE detected following LTACH admission, and the median days from admission to CRE detection in these patients (interquartile range) was 25 (11–43). Seven more patients were already positive for CRE at the time of LTACH admission. Molecular genotyping and MLST of 8 CRE isolates demonstrated that all isolates belonged to the same strain type (ST258) and contained the *bla*_KPC-3_ sequence.

**Conclusions:**

The majority of patients with CRE presented several days to weeks after LTACH admission, indicating possible organism acquisition in the LTACH itself. The genetic similarity of the CRE isolates tested could further indicate the occurrence of horizontal transmission in the LTACH or simply be representative of the regionally dominant strain.

The spread of Carbapenem-resistant Enterobacteriaceae (CRE) through acute care hospitals and long-term acute care hospitals (LTACHs) represents a major threat to the patients in the United States [1, 2]. *Klebsiella pneumoniae* Carbapenemase (KPC)–producing Enterobacteriaceae, an increasingly common type of CRE at LTACHs, express serine β-lactamases that are only sometimes susceptible to last-resort antimicrobials such as tigecycline, colistin, and gentamicin [1, 3] or to newer, expensive agents such as ceftazidime-avibactam [4]. Treatment with such agents is associated with significant drug-related toxicity and/or cost [5, 6]. Moreover, there is a lack of data from randomized trials to clearly demonstrate the efficacy of these agents in treatment of CRE infection [4, 7].

Risk factors for CRE include the presence of comorbidities and extensive and prolonged exposure to antibiotics and hospital-related invasive procedures [8]. As all aforementioned characteristics are typical of patients admitted to LTACHs, residents of these facilities are considered important reservoirs for multidrug-resistant organisms (MDROs), including CRE [9]. Investigators have reported that a recent stay at an LTACH is an important risk factor for CRE carriage [9]. Several surveillance studies conducted in the Chicago area reported that the prevalence of KPC-producing CRE in LTACHs was approximately 8–10 times higher than in acute care hospitals [10, 11]. Few studies have reported on the timing of CRE colonization and infection among patients residing in LTACHs. The aim of this project was to assess the timing of CRE detection in patients admitted to an LTACH. We also sought to investigate the genetic relatedness of CRE strains among the patients of 1 LTACH.

## METHODS

### Study Settings and Design

A retrospective study was conducted on patients infected or colonized with CRE who were admitted to a 77-bed free-standing LTACH in Detroit between January 1, 2011, and July 31, 2012. Colonization was defined as a positive CRE culture in a patient with no clinical signs or symptoms of infection. Infection was defined as the isolation of CRE from a sterile body site such as blood or isolation from a nonsterile site in a patient with clinical signs of infection. Diagnosis of CRE pneumonia required the presence of new or worsening chest infiltrate and purulent lower respiratory tract secretions at the time of CRE detection in sputum [12]. Urinary tract infections and wound infections were diagnosed if patients had localized signs/symptoms of infection or met systemic inflammatory response syndrome (SIRS) criteria at the time of CRE detection in urine and wounds, respectively, and no other etiology of SIRS was identified [13].

No active surveillance for MDROs including CRE was conducted at the LTACH during the study period; therefore, only CRE detected from cultures taken as part of routine clinical care in the LTACH were included. In addition, 7 patients who were known to be CRE-positive at the time of LTACH admission because of positive cultures at the hospital from which they were transferred were also included in the study cohort. LTACH policy directed that patients be placed in single rooms on contact precautions when CRE was detected in clinical cultures. Isolation measures in a patient were implemented for a minimum of 1 year since the last positive CRE culture. Dedicated equipment (such as blood pressure cuffs, stethoscope, thermometers) was also used while caring for CRE patients. No new infection control interventions were implemented during the study period. A registered nurse functioned as the LTACH’s full-time infection preventionist.

### Data Collection

Patients’ medical records were reviewed to abstract information pertaining to demographics and comorbid conditions, as well as the anatomic site and date of CRE culture. Other potential risk factors for CRE infections were assessed, including recent hospitalizations, exposure to antimicrobials, invasive procedures, and presence of indwelling devices. Data related to antibiotic use and hospital-related exposures were collected for the 30-day period before the culture date.

Incidence was expressed as number of unique episodes of CRE infection per 10 000 inpatient-days (ie, only the first episode of CRE infection in a patient was considered). To calculate the days from LTACH admission to detection of CRE, the date of admission to LTACH was counted as day 0 and the initial date that a positive culture was obtained was considered to be the CRE culture date. Thirty-day mortality rate was captured for all patients.

### Microbiology

All culture samples positive for Enterobacteriaceae were screened for Carbapenem resistance using the 2011 Clinical Laboratory Standards Institute (CLSI) guidelines for antimicrobial susceptibility testing [14]. Detection of Carbapenemase production was performed using the modified Hodge test [15]. To determine if there was a clonal relationship between the different CRE strains, molecular genotyping was carried out on 8 available CRE isolates. Molecular genotyping for detection of the *bla*_KPC_ gene was performed using real-time polymerase chain reaction (PCR) assay. Repetitive sequence–based PCR (rep-PCR) and multilocus sequence typing (MLST) were also performed to determine the clonal relationships among the 8 CRE isolates, similar to an earlier study [16].

### Statistical Analysis and Study Ethical Review

Analyses were performed using IBM SPSS Statistics 22 (SPSS Inc., Chicago, IL). In addition to descriptive statistical analyses, a time trend analysis was performed to describe the pattern of CRE detection over time. The current study was reviewed and approved by the facility ethics committee of the Wayne State University Institutional Review Board.

## RESULTS

### Incidence, Demographics, Clinical Characteristics, and Outcomes of the Cohort

The study cohort included 30 patients with CRE-positive cultures. There was a total of 35 112 inpatient-days during the study period, with an incidence of CRE infection of 6.83 episodes per 10 000 inpatient-days. Among the 30 patients, 29 patients were transferred to the LTACH from 14 different acute care hospitals, and 1 patient was transferred from an adult foster care home. Seven of 30 patients had positive CRE cultures before admission, among whom 3 had CRE infection and 4 were colonized with CRE.

The mean age of the cohort was 69 ± 12.41 years; 25 (83%) patients were African American, and 19 (63%) were female. The median Charlson’s score of our cohort (interquartile range [IQR]) was 5 (3.75–7). The top 5 comorbid conditions reported among our cohort included hypertension (86%), diabetes mellitus (70%), chronic obstructive pulmonary disease (57%), end-stage renal disease (55%), and congestive heart failure (54%).

During the 30-day period before positive CRE culture, 19 (63%) patients were ventilator-dependent, 6 (20%) patients had an indwelling central venous catheter, 8 (27%) had an indwelling urinary catheter, and 9 (30%) had undergone surgery. Twenty patients (67%) were treated with at least 1 antibiotic within the prior 30 days, among whom 19 (95%) had been exposed to 2 or more antibiotics. Twenty-one of 30 (70%) patients had overlapping stays in the same unit of the LTACH. Five patients died during their hospitalization within 30 days of the CRE culture date (30-day mortality rate = 17%), 4 (80%) of whom had bloodstream infection with CRE.

### Source of CRE Isolates and Time of Detection

**Figure 1.**
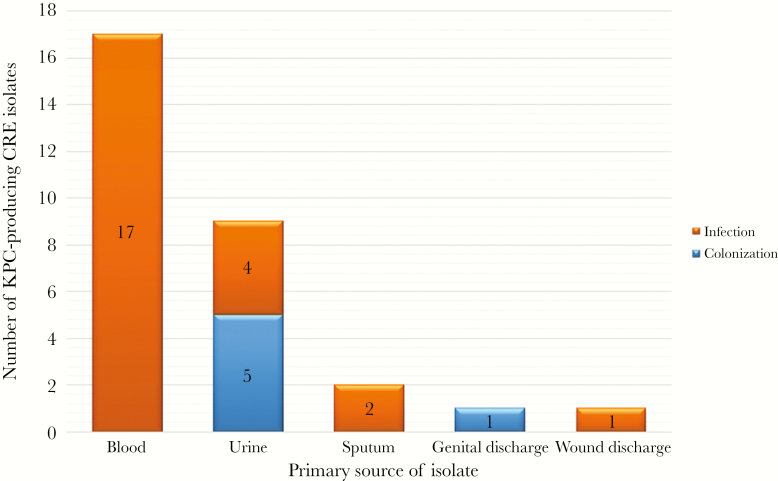
Distribution of colonization and infection among the various sources of CRE isolates. Abbreviations: CRE, Carbapenem-resistant Enterobacteriaceae; KPC, *Klebsiella pneumoniae* Carbapenemase.

Of all CRE-positive cultures, 24 (80%) represented CRE infection and 6 (20%) CRE colonization. The source of CRE samples in infected patients included 17 (71%) blood samples, 4 (17%) urine samples, 2 (8%) sputum samples, and 1 (4%) wound discharge sample (Figure 1). Five (83%) of the colonizing samples were isolated from urine, and 1 (17%) was isolated from genital discharge.

**Figure 2.**
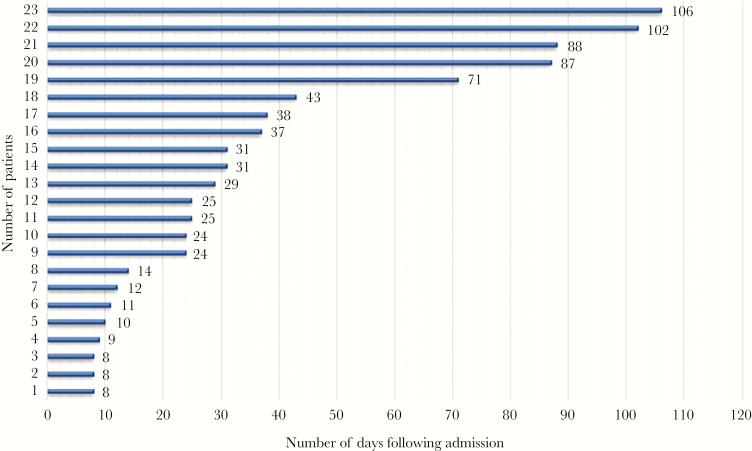
Time to culture positivity in patients developing CRE infection/colonization following LTACH admission. Abbreviations: CRE, Carbapenem-resistant Enterobacteriaceae; LTACH, long-term acute care hospital.

**Figure 3.**
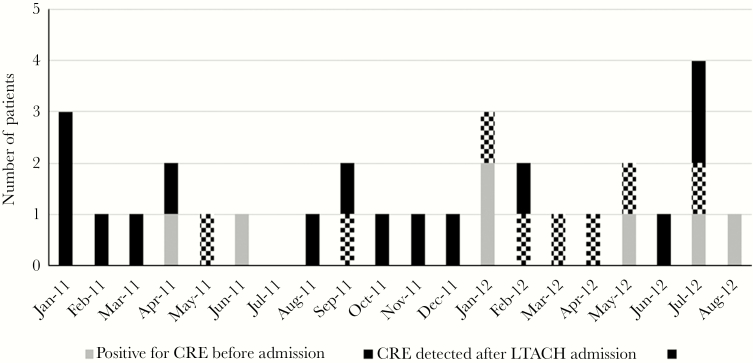
Distribution of CRE isolates during the study period. Checkered rectangles indicate CRE isolates detected after LTACH admission which underwent molecular genotyping. Abbreviations: CRE, Carbapenem-resistant Enterobacteriaceae; LTACH, long-term acute care hospital.

Twenty-three patients had CRE detected following admission to an LTACH. The median number of days from admission to CRE detection in these patients (IQR) was 25 (11–43) (Figure 2). The remaining 7 patients were already infected/colonized with CRE before LTACH admission (as mentioned above). The distribution of CRE isolates during the study period is given in Figure 3.

### Molecular Genotyping

**Figure 4.**
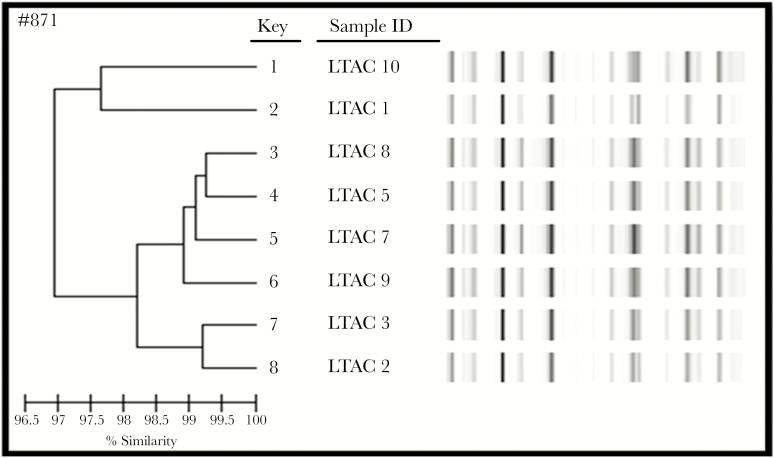
Molecular genotyping using repetitive sequence–based polymerase chain reaction of 8 Carbapenem-resistant Enterobacteriaceae isolates.

All 8 CRE isolates that underwent molecular genotyping were from patients who developed CRE infection following LTACH admission (Figure 3). These 8 patients were transferred from 7 different acute care hospitals. Molecular genotyping using rep-PCR demonstrated that the 8 CRE isolates shared >97% similarity and belonged to the same strain type. MLST demonstrated that all 8 isolates were ST258 strain type and contained the *bla*_KPC-3_ sequence (Figure 4).

## DISCUSSION

Patients in LTACHs have been identified as an important reservoir of CRE, and these facilities have been major drivers of regional CRE outbreaks [10, 17, 18]. Previous studies on LTACH patients have looked at factors associated with CRE colonization [2, 17, 19–21]. Our study evaluated 30 LTACH patients infected or colonized with CRE, and characteristics of infected and colonized patients were separately analyzed. In this study, LTACH patients infected with CRE were older adults, had multiple comorbidities, had exposure to multiple antibiotics, frequently had invasive devices, or had undergone recent surgery. These findings are similar to those reported in other studies [2, 17, 20].

The most striking finding of this study was that most CRE isolates were detected several days or weeks after LTACH admission, with 23 patients developing infection/colonization between 8 and 106 days after admission (median, 25 days) (Figure 2). This indicates that in many instances, patients likely acquired CRE during their LTACH stay. A previous study in 4 Chicago LTACHs showed that the median time to positive CRE rectal surveillance culture following a negative surveillance culture result (ie, the time to CRE acquisition) (IQR) was 16.5 (7.5–28.8) days when surveillance cultures were done at admission and biweekly thereafter [22]. The longer time to CRE detection in our study compared with this study was likely due to reliance on clinical cultures, which usually become positive many days to weeks after rectal colonization (ie, rectal CRE carriage precedes CRE infection) [23, 24]. Therefore, it can be hypothesized that some of our patients who acquired CRE during their LTACH stay went undetected due to lack of periodic active surveillance, only to be later identified when they developed infection.

On the other hand, some of these patients (in addition to patients who were already known to be colonized at the time of admission) might have been colonized for long periods before developing infection, and thus might have already been colonized with CRE when they were admitted to the LTACH. As active surveillance was not done at the time of admission, such “present on admission” CRE cases might have gone unnoticed, potentially leading to CRE spread within the LTACH. Identifying asymptomatically colonized patients early in their LTACH stay through active surveillance, followed by prompt institution of infection control measures for colonized patients, might have decreased opportunities for horizontal transmission. Such infection control measures, which have been proven to prevent CRE spread in the LTACH setting, include rigorous adherence to hand hygiene, contact precautions using gloves and gowns, use of private rooms for colonized patients or cohorting of colonized patients on dedicated floors or units, appropriate environmental cleaning, chlorhexidine bathing of patients, and use of dedicated equipment such as stethoscopes, blood pressure cuffs, thermometers [2, 19, 25].

Our study also demonstrated genotypic similarity between all 8 of the CRE isolates tested (all belonged to the ST258 strain type and contained the *bla*_KPC-3_ sequence). These 8 strains were evenly distributed during the study period and were identified in patients admitted from 7 different acute care hospitals. Although all this could indicate horizontal transmission, it could also simply be representative of the regionally dominant strain ST258 found in Michigan [26]. In addition, our patient cohort was admitted from 14 different acute care hospitals, and 70% of them had overlapping stays in the same unit of an LTACH. Admission from different acute care facilities and overlapping stays also suggest the presence of horizontal transmission (ie, spread from 1 patient to another, likely due to contaminated health care workers and/or environment) in the study LTACH.

One of the limitations of this study is its observational and retrospective nature, which precluded routine use of rectal swabs to detect asymptomatically colonized patients. Also, all CRE isolates were identified via clinical cultures due to suspected infections, which likely led to underestimation of CRE burden at the facility. We also had few isolates available for molecular genotyping; therefore, it is unclear if testing our entire cohort would have similarly identified a genotypically related strain. In addition, the study included a small number of CRE patients from a single LTACH located in an underserved area, which may limit the generalizability of the findings.

In conclusion, this study demonstrates that the majority of patients with CRE presented several days or weeks after LTACH admission, indicating potential acquisition and spread of CRE within the LTACH. Genetic relatedness of the tested CRE isolates could further indicate that horizontal transmission occurred, or it could simply be representative of the regionally dominant strain. These findings suggest that active surveillance (with rapid molecular diagnostic tests or using selective rectal cultures) should be considered among subjects at the time of LTACH admission, and possibly throughout their LTACH stay [25, 27]. In addition, communicating CRE colonization or infection status during transitions of care is likely to be helpful in preventing inter- and intrafacility spread. Implementing CRE-preventive strategies in LTACHs as recommended by the Centers for Disease Control and Prevention is urgently needed to contain their spread [28].
